# Cellular and Molecular Mechanisms Underlying the Strong Neonatal IL-12 Response of Lamb Mesenteric Lymph Node Cells to R-848

**DOI:** 10.1371/journal.pone.0013705

**Published:** 2010-10-28

**Authors:** Stéphanie Ferret-Bernard, Aude Remot, Sonia Lacroix-Lamandé, Coralie Metton, Nelly Bernardet, Françoise Drouet, Fabrice Laurent

**Affiliations:** Equipe «Contrôle et Immunologie des Maladies Entériques du Nouveau-Né», UR1282 Infectiologie Animale et Santé Publique, INRA Nouzilly, Nouzilly, France; University of Cambridge, United Kingdom

## Abstract

**Background:**

Comparative studies on the response of neonates and adults to TLR stimulation have been almost exclusively limited to comparisons of human neonatal cord blood cells with peripheral blood from adults, and analyses of spleen cell responses in mice. We need to extend these studies and gain further information regarding such responses at mucosal sites.

**Methodology/Principal Findings:**

We used sheep as a large animal model to study TLR agonist responses in the lymph nodes draining the intestine, an organ that must adapt to profound changes after birth. In response to the imidazoquinoline compound R-848, neonatal mesenteric lymph node (MLN) and spleen cells produced more IL-12 and, consequently, more IFNγ than their adult counterparts. This difference was age-related for both organs, but the preferential IL-12 response decreased more rapidly in the MLN, with young animals producing similar amounts of this cytokine to adults, from the age of 20 days onwards. Intracellular assays and depletion experiments identified CD14^+^CD11b^+^CD40^+^ cells as the main producer of IL-12. These cells accounted for a greater proportion of neonatal than of adult MLN cells, and also produced, in direct response to R-848, more IL-12 after isolation. This strong IL-12 response in neonates occurred despite the production of larger amounts of the regulatory cytokine IL-10 and the stronger upregulation of SOCS-1 and SOCS-3 mRNA levels than in adult cells, and was correlated with an increase in p38/MAPK phosphorylation.

**Conclusions/Significance:**

This is the first attempt to decipher the mechanism by which neonatal MLN cells produce more IL-12 than adult cells in response to the TLR8 agonist R-848. CD14^+^CD11b^+^CD40^+^ IL-12-producing cells were more numerous in neonate than in adult MLN cells and displayed higher intracellular responsiveness upon R-848 stimulation. This work provides relevant information for future vaccination or immunostimulation strategies targeting neonates.

## Introduction

Children and young animals are more vulnerable to infections because their immune system is still developing and differs in several ways from that of adults. This higher level of susceptibility has often been attributed to differences in the number, phenotype and/or impaired functions of cells from both innate and adaptive immune compartments. However, in certain circumstances, the innate immune cells of neonates may be more responsive to cytokine stimuli or microbial ligands [1]–[4]. It is therefore important to identify these specific features, with a view to developing new strategies for boosting the neonatal immune system to improve disease control.

In both humans and mice, neonates have a diminished capacity to mount the Th1-type responses involved in controlling intracellular pathogens [5]–[7]. TLRs are innate immune receptors involved in pathogen recognition and their agonists are widely used in vaccination and immunostimulation strategies for promoting Th1 polarisation [8]–[10]. Several studies have investigated the specific features of neonatal antigen-presenting cell (APC) responses to TLR ligands in mice and humans. In mice, these studies have been performed on spleen APCs, because the rates of recovery of APCs from other lymphoid organs and peripheral blood are low. It has been shown that neonatal splenic macrophages stimulated with various agonists of TLR-2, -4 and -9 produce fewer proinflammatory cytokines than adult cells, due to excessive IL-10 secretion [11], [12]. By contrast, isolated neonatal murine splenic CD11c^+^ DCs produce even more IL-12p70 than their adult counterparts after TLR stimulation [13]. In humans, stimulation of most of the TLRs on cord blood-derived monocytes and APCs results in the impaired production of various cytokines supporting Th1-type responses, including TNFα and IL-12, together with a strong production of IL-6, IL-10 and IL-23, polarising the immune response towards the Th17 and Th2 pathways [1], [14]–[16]. By contrast, TLR8 agonists seem to be uniquely effective to induce Th1-polarizing cytokine production by human neonatal APCs, making these agonists promising candidate adjuvants for enhancing the immune responses in newborns [1]. In ruminants, TLR8 agonists are also highly effective at inducing IFNγ release by peripheral blood monocytes [17]. Consistent with this finding, we observed that the TLR8 agonist R-848 induced a preferential Th1-type cytokine response in neonatal goat cells in intestinal draining lymph nodes [18], although the mechanism underlying this preferential response was not elucidated and merits further investigation. Large animal models, allowing the isolation of sufficient numbers of cells from newborn animals only a few days old, are of particular interest for studying TLR responses at mucosal sites.

In this study, we investigated the *in vitro* cytokine response of ovine mesenteric lymph nodes (MLN) and spleen cells isolated at different ages to the promising TLR8 synthetic agonist R-848. We found that neonatal cells produced more IL-12 than adult cells. This difference was age-related, with MLN and spleen cells displaying different response kinetics. CD14^+^ cells were identified as the main producers of IL-12, and the neonatal IL-12 secretion observed in response to R-848 was shown to be related to differences affecting both the proportions of these cells and the qualitative response of the downstream intracellular signalling pathway, involving, in particular, the level of p38/MAPK phosphorylation.

## Materials and Methods

### Animals and cell isolation

The Préalpes adult sheep, neonates (aged 6 to 14 days) and 20-day-old lambs used in this study were reared in conventional but protected sanitary facilities (PFIE, INRA, F-37380 Nouzilly, France). Newborn lambs were not separated from their mothers until one day after birth, to allow them to suckle colostrum, and were then fed *ad libitum* with reconstituted milk. Experimental protocols were designed in compliance with French law (Décret 2001-464 29/05/01) and EEC regulations (86/609/CEE) concerning the care and use of laboratory animals. Euthanasia was performed after electric stunning according to the AMVA guideline (2007) on euthanasia. Lymph nodes and spleen were removed from euthanasied animals at INRA-Nouzilly slaughterhouse with the consent from the slaughterhouse.Cells from freshly removed MLN or spleen were isolated as previously described [18]. Briefly, cells were isolated by mechanical dissociation of the tissue on 200 µm pore nylon gauze and then passed through a nylon mesh with 60 µm pores. Mononuclear cells were finally obtained after centrifugation over a Histopaque gradient.

### Reagents

PolyI:C, Resiquimod (R-848), Gardiquimod, CL075 and CL087 were purchased from Invivogen (Toulouse, France). PMA and ionomycin were purchased from Sigma. Recombinant human TGFβ1 was obtained from AbD Serotec (Oxford, UK). Recombinant ovine IL-12 and IL-10 were kindly provided by S. Wattegedera (Moredun Research Institute, Edinburgh, UK).

### TLR agonist stimulation and cytokine quantification by ELISA

MLN or spleen cells were stimulated, at a density of 1.5×10^6^/ml in complete RPMI 1640 medium (Gibco-Invitrogen, Cergy-Pontoise, France) supplemented with 10% FCS, 100 IU/ml penicillin, 100 µg/ml streptomycin sulfate and 50 µM β-mercaptoethanol (Merck Chemicals Ltd., Nottingham, UK) with polyI:C (12.5 µg/ml), R-848 (0.5 µg/ml), Gardiquimod (5 µg/ml), CL075 (0.5 µg/ml) or CL087 (0.5 µg/ml). Non-specific stimulation was performed with a mixture of PMA (50 ng/ml) and ionomycin (500 ng/ml). In some experiments, rhTGFβ1 or rovIL-10 was added to MLN cells at the same time as R-848. Culture supernatants were harvested and stored at -20°C until assayed for the detection of cytokines by ELISA. Intracellular staining for IL-12 was performed on MLN cells cultured in complete RPMI medium with or without R-848 for 8 h. Brefeldin A (Sigma) was added to the cells at a concentration of 5 µg/ml, for the last 5 h of culture. In the IL-12 neutralisation assay, mouse anti-bovine IL-12 (clone CC301, AbD Serotec) was added, at a concentration of 10 µg/ml, at the same time as the TLR agonists. In some experiments, the medium culture was supplemented with 10% neonatal autologous plasma or 10% adult plasma instead of 10% FCS as stated above. For p38/MAPK inhibition, 20 µM of SB203580 was added at the beginning of the culture.

Pairs of antibodies against bovine cytokines were obtained from AbD Serotec and have been shown to recognise ovine IL-12 [19], [20], IFNγ [21] and IL-10 [20], [22]. ELISA was carried out as previously described [18]. Antibody pairs used for IL-12 ELISA cannot distinguish between IL-12p70 and the IL-12p40 subunit but have been shown to both block the biological activity of the cytokine [19]. Incubations were performed at 37°C for IL-10 and at room temperature for the other ELISA.

### Cell sorting and flow cytometry

A high-speed MoFlo cell sorter (Dako, Trappes, France) was used to sort freshly isolated total MLN cells. In these experiments, cells were labelled with a mouse antibody specific for ovine CD14 (clone CAM36A, VMRD, Pullman, USA) and stained with a fluorochrome-conjugated goat anti-mouse immunoglobulin antibody (Caltag-Invitrogen). As CD14 displays intermediate levels of expression by lymph granulocytes in sheep [23], CD14^+^ cell sorting was always performed after gating on non-granulocytic MLN cells according to SSC/FSC analysis, although most granulocytes were discarded on the Histopaque gradient. Both CD14^+^ and CD14^−^ cell fractions were cultured *in vitro* at a density of 1.5×10^6^/ml (3.10^5^/well), with and without R-848, or were directly analysed by qRT-PCR.

For the intracellular staining of IL-12, surface staining was first carried out with the following purified monoclonal antibodies directed against ovine markers: CD11b (clone MM12A, VMRD), CD14 (clone CAM36A, VMRD), MHC class II (clone 28.1, AbD Serotec) and CD205 (clone CC98, AbD Serotec). Fixed and permeabilised cells were then incubated with the mouse anti-bovine/ovine IL-12 antibody (clone CC301, AbD Serotec).

For characterisation of the CD14^+^ cell phenotype, total MLN cells were double-labelled with an anti-ovine CD14 (clone TÜK4, AbD Serotec) and an anti-CD11b (clone MM12A) or anti-CD11c (clone BAQ153A) antibody (both purchased from VMRD), or antibodies against CD26 (clone CC69), CD205 (clone CC98), MHC class II molecules (clone 28.1) (all from AbD Serotec), or against CD206 (clone 3.29B1.10, Beckman Coulter, Villepinte, France) or CD40 (clone ILA156 originally produced by J. Naessens (ILRAD, Nairobi, Kenya)). Flt3 (CD135) expression was determined by incubating cells with a His_6_-tagged porcine Flt3 ligand, and labelling with an anti-His_6_-tag monoclonal antibody, as previously described [24].

Every staining reaction described in this section was followed by further incubation with fluorochrome-conjugated goat anti-mouse immunoglobulin isotype antibodies (Caltag-Invitrogen). Isotype-matched antibodies were used as controls. Cells were finally analysed with a FACS-Calibur™ flow cytometer (BD Instruments, San Jose, USA) equipped with CellQuest Pro® software.

### RNA isolation and real-time RT-PCR

RNA was extracted with the NucleoSpin RNA II kit (Macherey-Nagel, Hoerdt, France), according to manufacturer's instructions and quantified using Nanodrop (Thermo Fisher Scientific, Courtaboeuf, France). Purified RNA was reverse-transcribed using oligo(dT) primers and M-MLV reverse transcriptase (Promega, Charbonnières-les-Bains, France). Couples of primers were designed on different exons using Primer 3 software to avoid DNA amplification (Table S1) and for each couple of primers, all qPCRs displayed efficiency between 90% and 110%. Diluted cDNA was combined with primers and IQ SYBRGreen Supermix (Bio-Rad, Hercules, USA) according to the manufacturer's recommendations and real-time assays were run on a Bio-Rad Chromo 4 (Bio-Rad). The specificity of the qPCR reactions was assessed by analysing the melting curves of the products and size verification of the amplicons. To minimise sample variations, we used identical number of cells and high quality RNA. Hypoxanthine phosphoribosyltransferase (HPRT) mRNA levels were used to normalise RNA quantification.

### Electron microscopy

Sorted CD14^+^ cells were fixed, post-fixed and dehydrated in a graded series of ethanol solutions. Cell pellets were embedded in Epon resin (Sigma). Ultrathin sections were cut, stained and deposited on electron microscopy grids coated with collodion membrane for examination under a Jeol 1010 transmission electron microscope (Jeol, Croissy-sur-Seine, France). For scanning electron microscopy, sorted CD14^+^ cells were incubated overnight in complete RPMI medium on glass coverslips. After fixation and post-fixation processes, samples were dehydrated in graded acetone solutions, dried to the critical point under CO_2_ and coated by gold sputtering before examination in an FEG Gemini 982 scanning electron microscope (Carl Zeiss, Nanterre, France).

### p38/MAPK phosphorylation analysis by western blotting

Sorted MLN CD14^+^ cells were incubated overnight, at a density of 1.5×10^6^/ml, in complete RPMI medium and were then starved by incubation for 2 h in RPMI alone. They were collected after stimulation with R-848 for 15 or 30 minutes, or as unstimulated cells. We resolved the lysates of 1×10^6^ cells on a 12% acrylamide gel by SDS-PAGE and transferred the resulting bands onto nitrocellulose membranes. The saturated blots were probed overnight with a primary rabbit IgG antibody directed against phosphorylated (Thr180/Tyr182) human p38/MAPK (1/1000, Cell Signaling Technology-Ozyme, Saint Quentin en Yvelines, France) and were then incubated with horseradish peroxidase-conjugated anti-rabbit IgG (1/2000, Cell Signaling Technology-Ozyme). The specific signals were visualised by chemiluminescence, with the Lumiglo® substrate system (Cell Signaling Technology-Ozyme). Blots were then stripped and reprobed as described above, but with a primary rabbit polyclonal IgG specific for human total p38/MAPK (1/1000, Cell Signaling Technology-Ozyme). Phosphorylated p38/MAPK (pp38) levels were quantified relative to total p38/MAPK protein levels, with Scion software.

## Results

### Strong IL-12 and IFNγ response of neonatal MLN cells in response to TLR agonists

**Figure 1:**
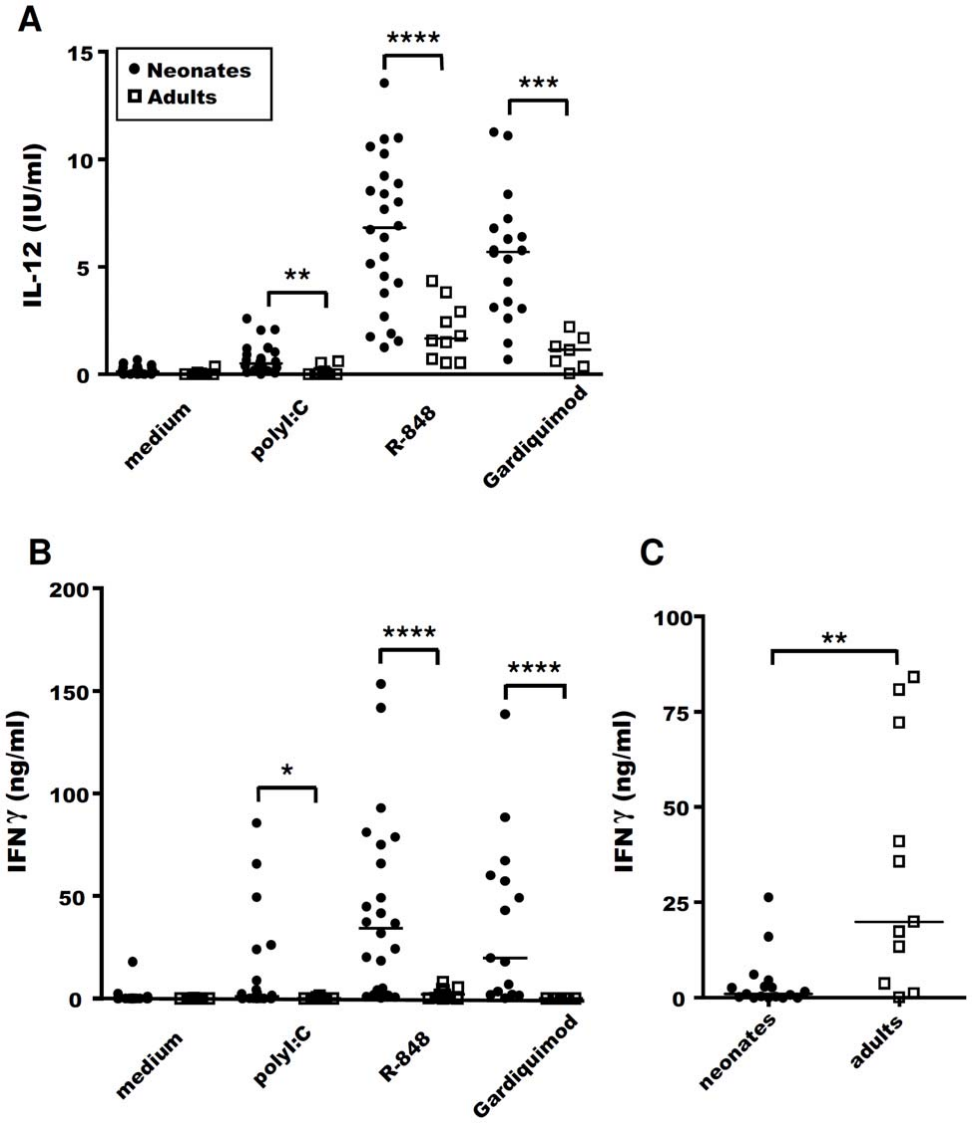
Comparative IL-12 and IFNγ responses of neonatal and adult MLN cells following TLR agonist stimulation. Neonatal (closed circles) and adult (open squares) MLN cells were cultured *in vitro* with or without 12.5 µg/ml polyI:C, 0.5 µg/ml R-848 or 5 µg/ml Gardiquimod. Supernatants were harvested after 48 h of culture and ELISA was carried out for IL-12 (**A**) and IFNγ (**B**) secretion. Non-specific cell stimulation was also carried out with 50 ng/ml PMA combined with 500 ng/ml ionomycin, with the supernatants assayed for IFNγ detection 48 h later (**C**). Each circle or square represents one neonate or one adult, respectively. Medians are shown for each stimulus. Non-parametric Mann-Whitney tests were used to compare data for neonates and adults: *p≤0.01; **p≤0.005; ***p≤0.001; ****p≤0.0005.

Various cytokine responses to TLR agonists have been described in cells isolated from the systemic compartment [1], [25]–[27], but these responses have not been studied in cells isolated from lymph nodes draining the intestinal mucosa with exception to our previous work on caprines [18]. We continued this work in ovine for practical reasons to further dissect the cellular mechanism involved in TLR agonist response in MLN. Cells isolated from neonatal and adult sheep were stimulated *in vitro* with polyI:C (TLR3 agonist), Resiquimod (also called R-848) and Gardiquimod (both agonists of TLR7/8) for 48 h, and cytokine levels were determined by ELISA. Neonatal MLN cells produced variable amounts of IL-12 (Fig. 1A) and IFNγ (Fig. 1B), but the amounts produced were significantly larger than those produced by adult MLN cells, with R-848 and Gardiquimod being the best inducers. However, adult MLN cells were not intrinsically deficient for IFNγ production compared to neonatal cells, as they produced significantly larger amounts of this cytokine in response to non-specific stimulation with PMA associated with ionomycin *in vitro* (Fig. 1C). Moreover, by neutralising the IL-12 *in vitro*, we showed that IL-12 made a major contribution to IFNγ production by neonatal cells (73±9% inhibition, p<0.05). The IL-12 ELISA used cannot distinguish between IL-12p70 and the p40 chain, however this data suggests that MLN cells of neonates produce more bioactive IL-12 in response to R-848. We therefore investigated the particular features of the neonatal cells producing IL-12 in our model.

### Neonatal cells highly expressed IL-12p40, IL-12p35 and IL-23p19 chains upon R-848 stimulation

**Figure 2:**
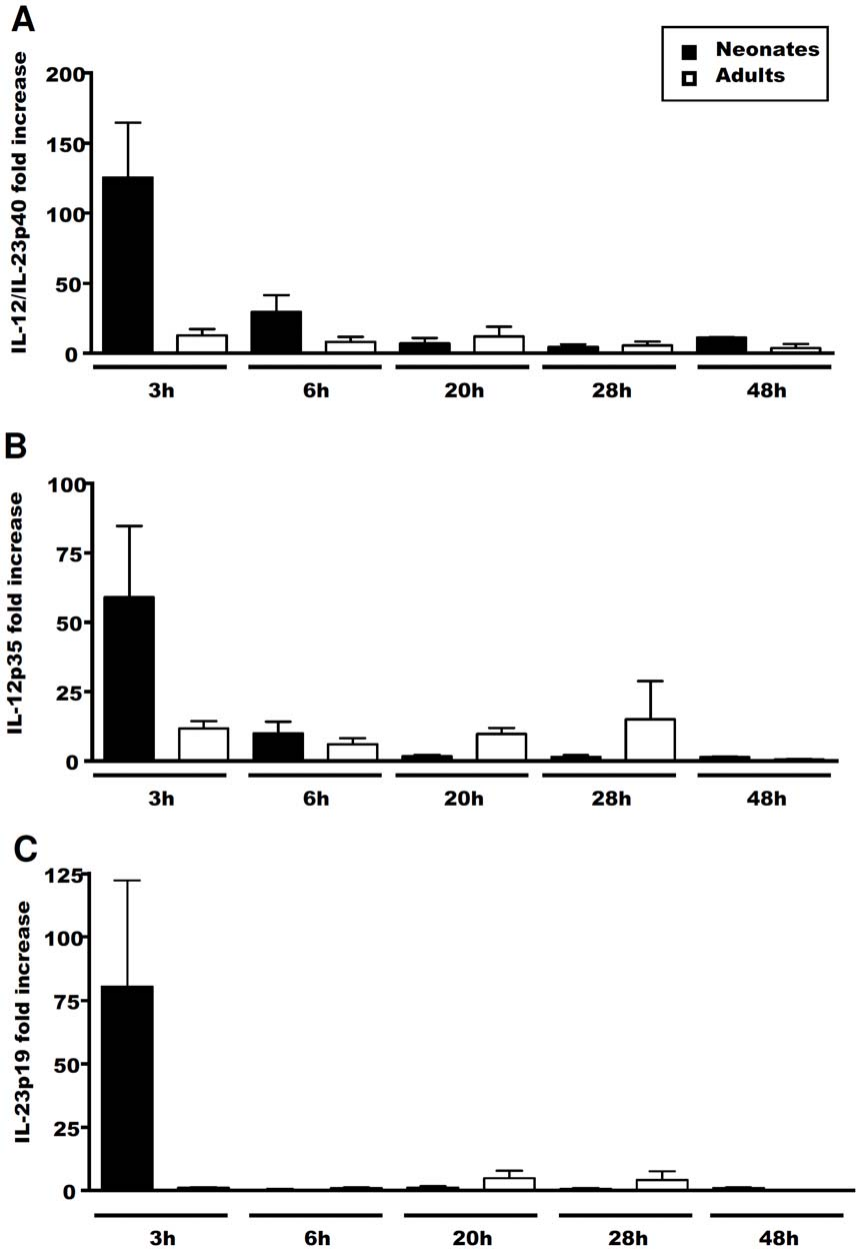
Levels of mRNA for IL-12/IL-23p40, IL-12p35 and IL-23p19 chains in MLN cells after R-848 stimulation. Neonate (black bars) and adult (white bars) MLN cells were cultured *in vitro* with or without 0.5 µg/ml R-848. At each time point, cells were recovered and mRNA levels for IL-12/IL-23p40 (**A**), IL-12p35 (**B**) and IL-23p19 (**C**) chains were determined by quantitative RT-PCR. After normalisation with the HPRT housekeeping gene, the data are shown as mean ± SEM values for the ratio of stimulated to resting cells.

IL-12 and IL-23 are heterodimeric cytokines of the IL-12 family that share the p40 chain. Defective IL-12 production, associated with an increase in IL-23 production, has been reported in human neonatal cells stimulated with TLR ligands [15]. As no ELISA for IL-23 is yet available for sheep, we investigated the levels of mRNA for the IL-12- and IL-23-specific chains, p35 and p19, respectively, and those for their common chain, p40, by quantitative RT-PCR, to identify putative differences in the expression of these cytokines between adults and neonates. We decided to focus on the TLR agonist R-848, as this molecule was the strongest inducer of IL-12 *in vitro*. Consistent with our ELISA findings for IL-12 (Fig. 1), qRT-PCR on MLN cells stimulated with R-848 showed that IL-12p40 and IL-12p35 mRNA levels were about 50 and four times higher, respectively, in neonatal cells than in their adult counterparts, 3 h after stimulation (Fig. 2A, 2B). Strong IL-23 mRNA expression was observed only in neonates (Fig. 2C), suggesting that, in response to R-848 stimulation, neonatal MLN cells produced not only more IL-12, but also possibly more IL-23, than did adult cells.

### The stronger IL-12 response of neonatal MLN cells rapidly weakens with age

**Figure 3:**
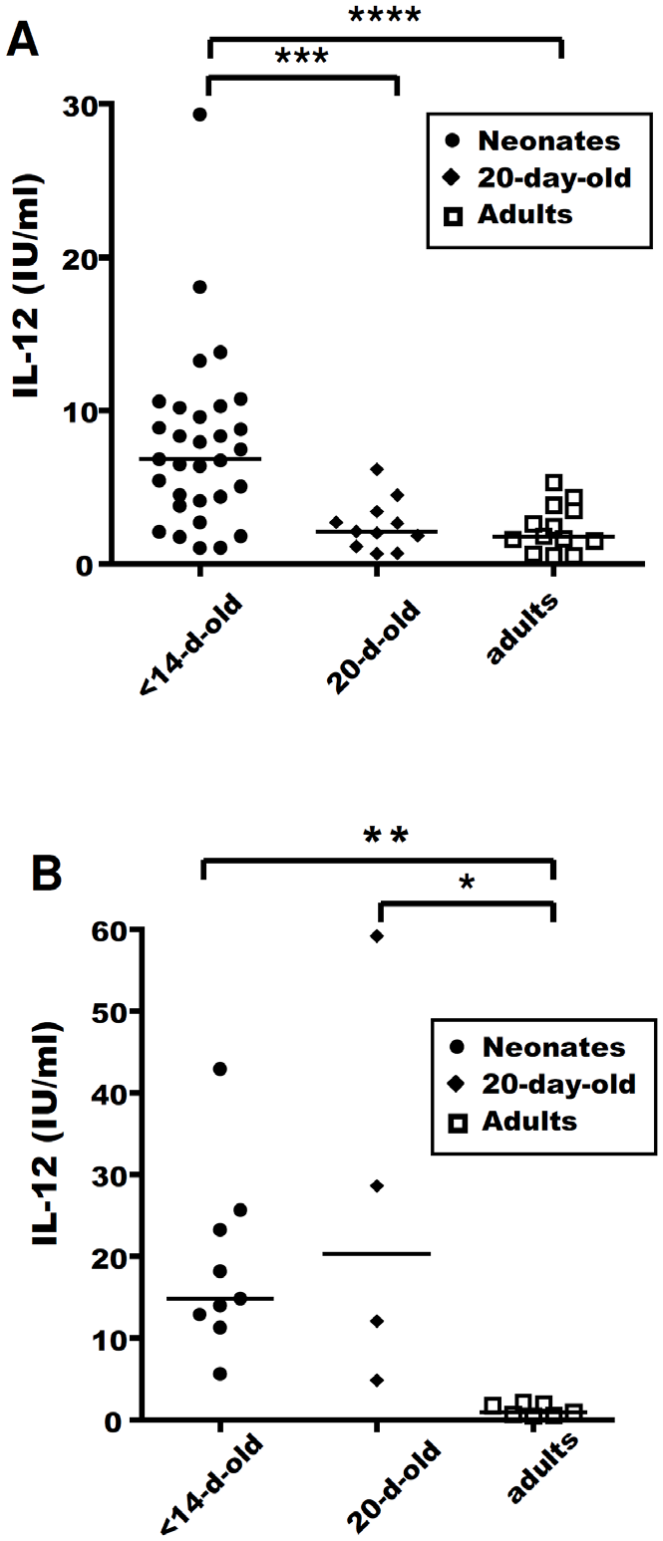
MLN and spleen cell IL-12 responses to R-848 stimulation as a function of age. MLN and spleen cells from 6- to 14-day-old neonates (closed circles), 20-day-old lambs (closed diamonds) and adults (open squares) were cultured *in vitro* for 48 h, with or without 0.5 µg/ml R-848. At the end of the culture period, supernatants were harvested and ELISA carried out to assess IL-12 secretion by MLN cells (**A**) or spleen cells (**B**). Medians are shown for each stimulus. Non-parametric Mann-Whitney tests were used to compare data for neonates, 20-day-old lambs and adults: *p≤0.01; **p≤0.005; ***p≤0.001; ****p≤0.0005.

We investigated whether the stronger production of IL-12 by neonatal MLN cells evolved rapidly with age, by comparing the IL-12 secretion of MLN cells from 20-day-old lambs with that of neonatal lambs and adult sheep. We found that, in response to *in vitro* R-848 stimulation, the initially high level of IL-12 secretion by neonatal MLN cells rapidly decreased as the animal aged, with cells from 20-day-old lambs producing as little IL-12 as adult cells (Fig. 3A). By contrast, the IL-12 secretion of splenocytes isolated from 20-day-old lambs, although quantitatively variable, remained significantly stronger than that of adult cells (Fig. 3B). Thus, the higher IL-12 response began to decline earlier in MLN cells than in spleen cells, suggesting major changes in TLR responsiveness in the lymph nodes within the first few days of life. Furthermore, the strong IL-12 response of neonatal cells was detected not only in the intestinal draining lymph nodes, but also in the systemic compartment.

### Strong TLR-induced IL-12 responses in neonates was not associated with regulatory cytokines TGFβ1 and IL-10

**Figure 4:**
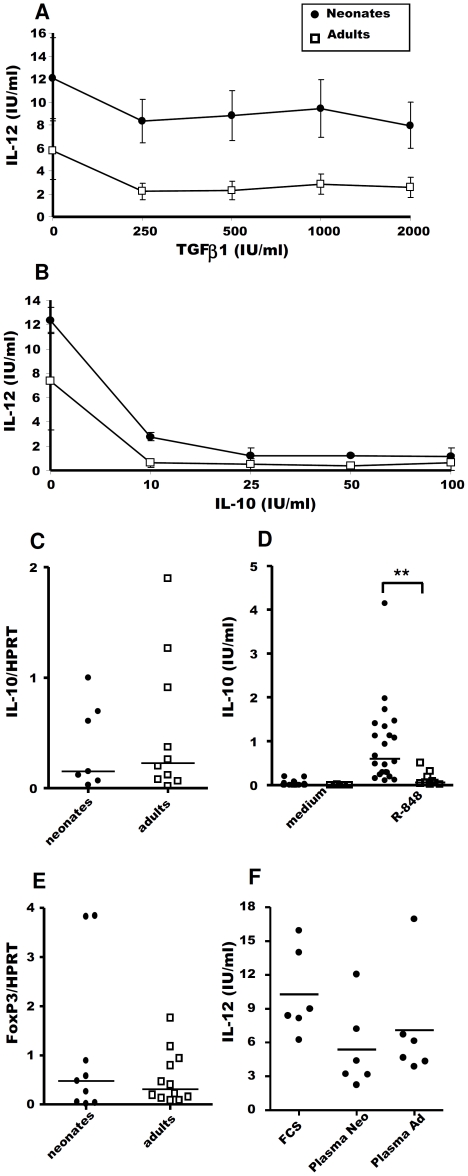
Roles of TGFβ1 and IL-10 in regulating IL-12 responses to R-848. MLN cells from neonates (closed circles) and adults (open squares) were cultured *in vitro* for 48 h, with or without 0.5 µg/ml R-848, in the presence of rhTGFβ1 (n = 7) (**A**) or rovIL-10 (n = 5) (**B**). Supernatants were harvested and ELISA was carried out to assess IL-12 secretion. The mean ± SEM level of IL-12 secretion is shown (**A**, **B**). RNA was extracted and purified from freshly isolated MLN cells. IL-10 mRNA levels were determined by quantitative RT-PCR. Median normalised values are presented for neonates (closed circles) and adults (open squares) (**C**). MLN cells from neonates (closed circles) and adults (open squares) were stimulated *in vitro* for 48 h with or without 0.5 µg/ml R-848. Supernatants were harvested and ELISA carried out to assess IL-10 secretion. Medians are indicated. Non-parametric Mann-Whitney tests were used to compare data for neonates and adults: **p≤0.001 (**D**). RNA was extracted and purified from freshly isolated MLN cells. Foxp3 mRNA levels were determined by quantitative RT-PCR. Median normalised values are presented for neonates (closed circles) and adults (open squares) (**E**). Comparison of the IL-12 response of neonate MLN cells to R-848 stimulation in culture medium supplemented with 10%FCS, 10% neonate autologous plasma or 10% adult plasma. Paired t-test between neonate and adult plasma were non-significant (**F**).

We investigated the effect of two regulatory cytokines widely expressed in the lymphoid tissues associated with the intestine, TGFβ1 and IL-10, on IL-12 production by neonatal and adult MLN cells. rhTGFβ1, despite its cross-reactivity and its suppressive effect on ovine cells (data not shown), had no significant effect on the response to R-848 (Fig. 4A). By contrast, IL-10 had a regulatory effect, as at a concentration of 25 IU/ml, it almost completely abolished IL-12 secretion by the MLN cells of both neonates and adults (Fig. 4B). We therefore investigated whether IL-10 in adult was produced in larger amounts in the intestine or MLN, potentially accounting for lower IL-12 response observed to R-848 stimulation. However, at homeostasis, IL-10 mRNA levels were similar in the MLN (Fig. 4C), such as for jejunum and Peyer's patch samples (data not shown). Following stimulation *in vitro* with R-848, neonatal cells produced low quantities but significantly more IL-10 than adult cells (Fig. 4D). Thus, despite the presence of detectable, but low levels, of IL-10 after R-848 stimulation, neonatal MLN cells produced more IL-12 than adult MLN cells. Regulatory T cells (Treg) are known to produce regulatory cytokines like IL-10 and TGFβ. To date, no antibody is available in sheep to characterise this cell population with the specific marker Foxp3. We therefore investigated the expression level of the transcription factor Foxp3 by real-time RT-PCR in neonate and adult MLN cells but found no significant difference in the level of expression (Fig 4E). Levy *et al*. observed that newborn plasma conferred substantially reduced BLP-, LPS-, and imiquimod-induced TNF-α release by adult monocytes [28]. However, it has to be noticed that the same authors did not observed similar effect of neonatal plasma on R-848-induced TNF-α release [28]. These results suggest that soluble factors in the plasma present in different concentrations in neonates and adults could affect differentially the IL-12 response. We therefore analysed the response of neonate MLN cells to R-848 in vitro in presence of 10% autologous plasma or 10% adult plasma. The IL-12 levels were reduced in 10% plasma compared to 10% FCS, but we did not observed any significant difference in the IL-12 levels released by neonatal cells cultivated with 10% neonate or adult plasma (Fig 4F).

### CD14^+^ and CD11b^+^ cells make a major contribution to IL-12 secretion in response to R-848

**Figure 5:**
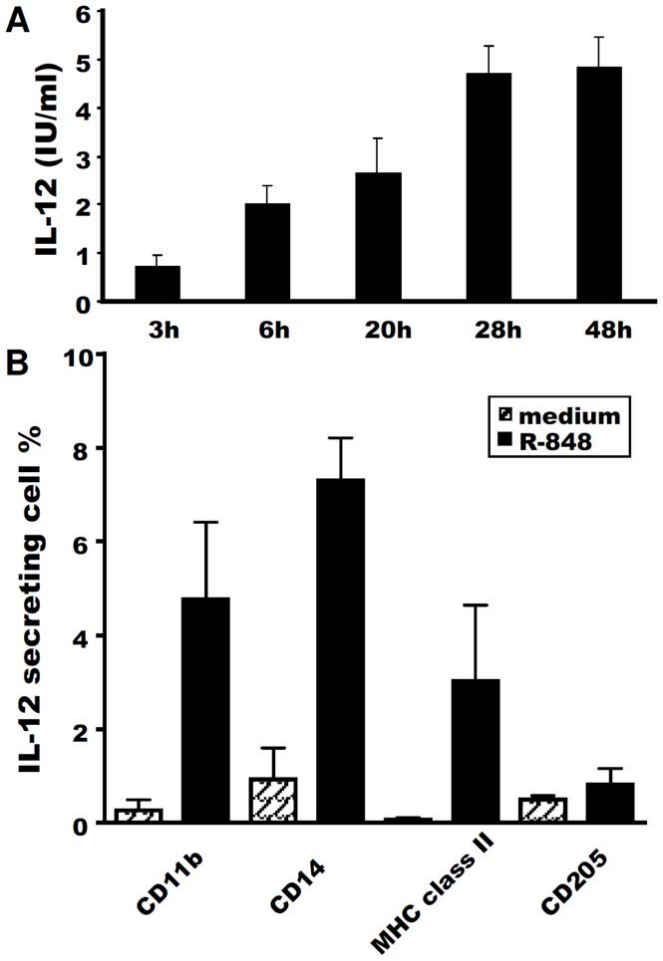
Main cell population producing IL-12 in response to R-848. Neonatal MLN cells were cultured *in vitro* with or without 0.5 µg/ml R-848 and, at various time points, supernatants were harvested and ELISA was carried out for IL-12. Mean ± SEM IL-12 secretion is shown for each time point (**A**). Neonatal MLN cells were cultured *in vitro* with (black bars) or without (hatched bars) 0.5 µg/ml R-848 for 8 h, with brefeldin A added for the last 5 h. Cells were harvested for IL-12 intracellular staining combined with labelling of CD11b, CD14, MHC class II and CD205. Three independent experiments were carried out and the data shown are the mean proportions of IL-12^+^ cells after gating on CD11b^+^, CD14^+^, MHC class II^+^ or CD205^+^ MLN cells (**B**).

For a more precise characterisation of the mechanisms governing these differences in IL-12 secretion between neonates and adults, we needed to identify the principal cell population responsible for secreting this cytokine in the neonatal MLN. ELISA kinetic assays showed that IL-12 secretion by neonatal MLN cells was induced rapidly, as it was detected as soon as 3 h after R-848 stimulation, with this cytokine continuing to accumulate until 28 h after stimulation (Fig. 5A), consistent with the findings for IL-12p40 and p35 mRNA levels (Fig. 2). We therefore added brefeldin A to neonatal MLN cells 3 h after stimulation with R-848 and incubated the cells with this compound for 5 h. Cells were then harvested for IL-12 intracellular staining combined with the labelling of several potential APC markers in the ovine model. Both CD14^+^ cells and CD11b^+^ cells made major contributions to IL-12 secretion in response to R-848 (Fig. 5B).

### CD14^+^ cells co-express CD11b and CD40 and have long dendritic processes

We characterised the cell population expressing IL-12 further, by double staining neonatal CD14^+^ cells from the MLN (Table 1). Most of the CD14^+^ cells also expressed CD11b. Moreover, all CD11b^+^ cells co-expressed CD14, accounting for the similar proportions of IL-12-secreting cells in each population. We therefore decided to focus our study on CD14^+^ cells. Most MLN CD14^+^ cells expressed MHC class II molecules, half expressed CD11c and only one fifth expressed CD205. Most of the CD14^+^ cells also co-expressed CD40 but CD135 (Flt3) was only weakly expressed.

**Table 1:** Phenotypic characterisation of neonatal CD14^+^ MLN cells.

CD11b^+^	CD11c^+^	CD26^+^	CD40^+^	CD135^+^	CD205^+^	CD206^+^	MHC-II^+^
81.1±4.0	47.3±1.9	2.7±0.1	82.7±1.5	8.9±0.9	21.4±1.2	38.6±0.5	62.1±5.6

Cell surface marker expression by CD14^+^ cells (%).

Neonatal MLN cells were double-labelled with a primary mouse antibody cross-reacting with CD14 and another antibody cross-reacting with or specific for sheep cell-surface markers: CD11b, CD11c, CD26, CD40, CD135, CD205, CD206 or MHC class II molecules. The table shows mean ± SEM percentages of cells positive for the various markers tested among the gated CD14^+^ cells. Three independent experiments were performed.

**Figure 6:**
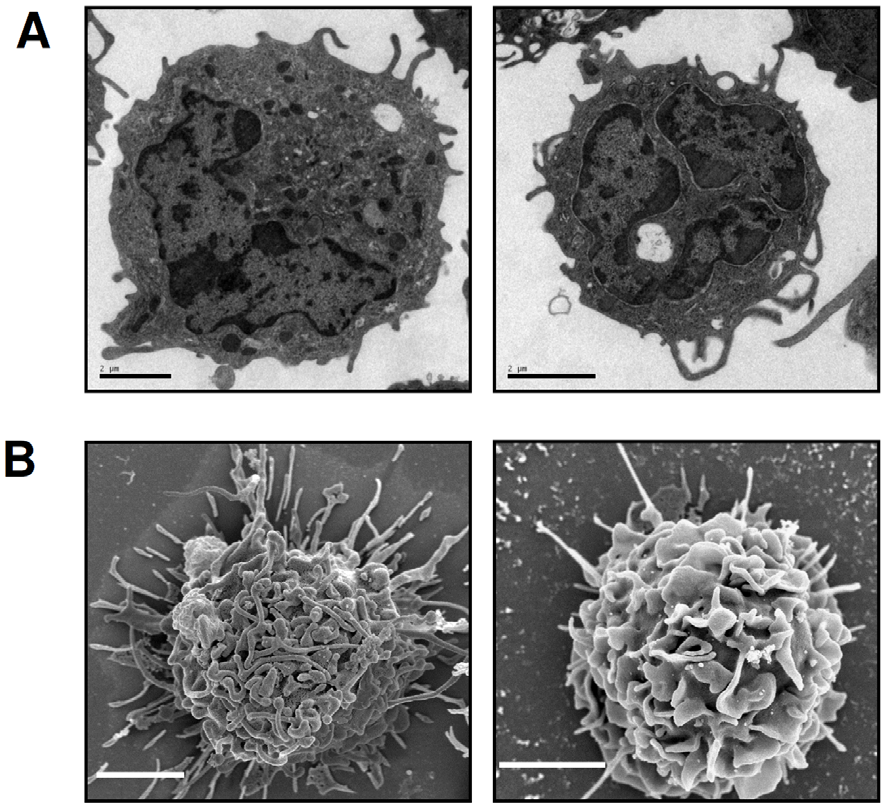
CD14^+^ morphology on microscopy. Two representative pictures of neonatal CD14^+^ MLN cells observed by transmission electron microscopy (**A**, **top panels**). Two representative pictures of scanning electron microscopy after overnight culture of neonatal CD14^+^ MLN cells on glass coverslips (**B**, **bottom panels**). The scale bars on the representative pictures indicate 2 µm.

Transmission electron microscopy demonstrated that neonatal CD14^+^ MLN cells had numerous processes and horseshoe-shaped nuclei, as expected for cells of the myeloid lineage (Fig. 6A). We also observed several dark electron-dense granules and a well developed endosomal compartment resembling that of ovine monocyte-derived DCs [29] and a CD11b^int/high^ subpopulation of bone marrow-derived DCs [30]. Scanning electron microscopy revealed that neonatal CD14^+^ MLN cells had a classical DC architecture, including a highly convoluted cell membrane and long dendritic processes (Fig. 6B).

### CD14^+^ MLN cells differ quantitatively and qualitatively from those of adults

**Figure 7:**
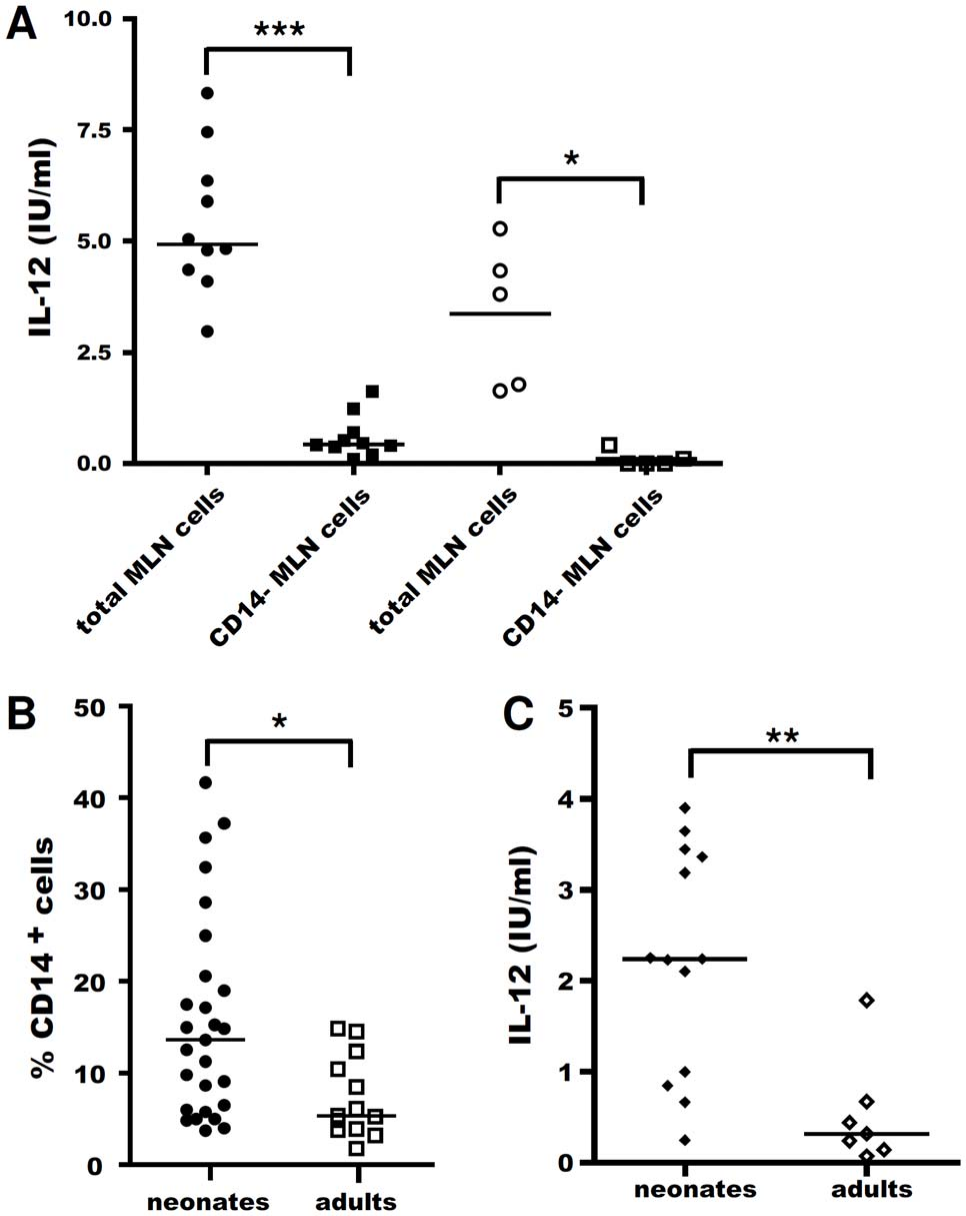
Role of CD14^+^ cells in IL-12 secretion in response to R-848 stimulation. Neonatal (closed circles) and adult (open circles) total MLN cells and CD14^−^ cells (closed and open squares, respectively) were cultured *in vitro* for 48 h with 0.5 µg/ml R-848. Supernatants were harvested and ELISA was carried out to assess IL-12 secretion. Paired t-tests were used: *p≤0.01; ***p≤0.0001 (**A**). The percentage of CD14^+^ cells in 6- to 14-day-old neonatal (closed circles) and adult (open squares) MLN cells was assessed by single staining and flow cytometry (**B**). Sorted CD14^+^ MLN cells from 6- to 14-day-old neonates (closed diamonds) or adults (open diamonds) were cultured *in vitro* for 48 h with 0.5 µg/ml R-848. Supernatants were harvested and ELISA was carried out to assess IL-12 secretion (**C**). Non-parametric Mann-Whitney tests were used to compare data for neonates and adults: *p≤0.01; **p≤0.005 (**A–C**).

We investigated the role of CD14^+^ cells in IL-12 secretion, by selecting CD14^−^ cells from adult and neonatal MLN cells, by flow cell sorting, before *in vitro* stimulation with R-848 for 48 h (Fig. 7A). CD14^−^ MLN cells secreted significantly less IL-12 than total MLN cells, not only in neonates (88±3% inhibition) but also in adults (98±1% inhibition), demonstrating the essential role of CD14^+^ MLN cells in both groups.

Having clearly defined CD14^+^ cells as the major population of IL-12-secreting cells among neonatal and adult MLN cells, we addressed the question of a possible quantitative defect affecting adult CD14^+^ cells. Indeed, flow cytometry analysis revealed that the proportion of CD14^+^ MLN cells in neonates was twice that in adults (15.8±2.1% versus 7.3±1.2%; Fig. 7B). However, the mean fluorescence intensity analysis revealed that CD14 was expressed at a similar level at the surface of adult and neonate CD14^+^ cells (data not shown).

We cultured the same number of neonatal and adult sorted CD14^+^ cells separately, in the presence of R-848, for 48 h. Neonatal CD14^+^ cells secreted IL-12 directly in response to R-848 (Fig. 7C), whereas adult cells displayed a significant defect in secretory activity, although they were able to secrete some IL-12 directly. Thus, quantitative and qualitative differences affecting CD14^+^ cells from neonates and adults were responsible for the strong neonatal IL-12 response to R-848.

### Higher levels of p38/MAPK phosphorylation in neonatal CD14^+^ cells stimulated with R-848

**Figure 8:**
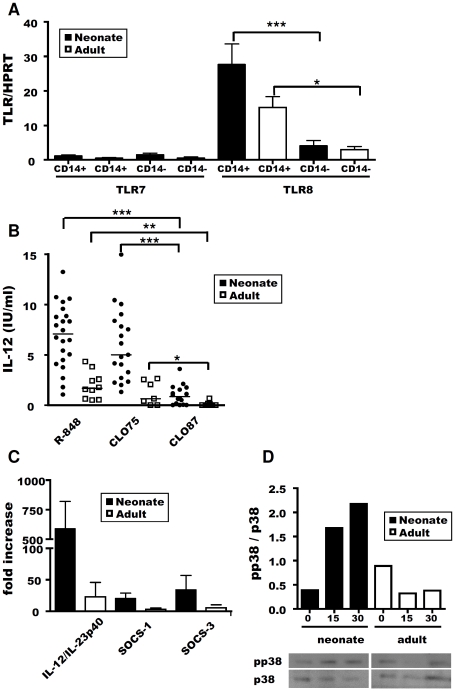
Intracellular signalling upon TLR8 ligation: p38/MAPK phosphorylation. RNA was extracted and purified from sorted CD14^+^ and CD14^−^ MLN cells from neonates (closed bars) and adults (open bars). TLR7 and TLR8 mRNA levels were determined by quantitative RT-PCR. Mean ± SEM normalised values for TLR expression are shown. Paired t-tests were used to compare TLR7 and TLR8 expression by CD14^+^ and CD14^−^ MLN cells: *p≤0.05; ***p≤0.001 (**A**). MLN cells from neonates (closed circles) and adults (open squares) were cultured *in vitro* for 48 h, with or without 0.5 µg/ml R-848, CL075 or CL087. Supernatants were harvested and ELISA was carried out for IL-12. Medians are shown for each stimulus. Paired t-tests were used to compare IL-12 secretion to different TLR agonists for both neonates and adults: *p≤0.05; **p≤0.005; ***p≤0.0001 (**B**). RNA was extracted and purified from sorted CD14^+^ MLN cells from neonates (black bars) and adults (white bars), 3 h after stimulation with R-848. IL-12/IL-23p40, SOCS-1 and SOCS-3 mRNA levels were determined by quantitative RT-PCR, as described above (**C**). Lysates of 1×10^6^ CD14^+^ MLN cells from neonates (n = 4) and adults (n = 3) were analysed, after 15 or 30 minutes of stimulation with R-848, by western blotting for pp38 and total p38. Representative images are shown, with corresponding quantification of pp38 with respect to total p38 protein (**D**).

We then investigated possible differences between neonates and adults in terms of TLR expression on the surface of CD14^+^ cells. The recognition of R-848 by human immune cells is mediated by both TLR7 and TLR8 [31], whereas the recognition of this molecule by murine immune cells involves only TLR7 [32]. We showed, by qRT-PCR analysis, that TLR7 mRNA levels were very low, and similar in CD14^+^ and CD14^−^ cells, in both neonates and adults (Fig. 8A). By contrast, TLR8 mRNA levels were higher in CD14^+^ cells than in CD14^−^ cells, in both groups. However, no significant difference in TLR8 mRNA levels was found between neonates and adults, suggesting that this difference resulted from TLR signalling.

We therefore stimulated total MLN cells isolated from neonates and adults with CL075, which mediates stimulation preferentially through TLR8, or with CL087, which mediates stimulation exclusively through TLR7, to identify the TLR used in small ruminant species more precisely. Neonatal MLN cells secreted more IL-12 than adult MLN cells in response to R-848, but also in the presence of CL075 and CL087 (Fig. 8B). However, both groups sensed CL075 similarly to R-848 and significantly better than CL087, indicating a preferential role of TLR8 rather than TLR7 in the recognition of R-848 by ovine immune cells.

We next analysed the levels of SOCS-1 and SOCS-3, two proteins shown to regulate TLR signalling indirectly in innate immune cells [33]. We found that the levels of mRNA encoding SOCS-1 and SOCS-3 were upregulated as soon as 3 h after stimulation with R-848 in neonatal MLN cells, but not in adult MLN cells (data not shown). These findings were confirmed in neonatal and adult CD14^+^ cells (Fig. 8C). SOCS-1 and SOCS3 proteins could be produced to regulate R-848 response but this could not explain the difference in IL-12 response between neonates and adults. We therefore analysed the phosphorylation of p38/MAPK, which has been identified as a key signalling molecule downstream from R-848/TLR8 activation. We also confirmed p38 MAPK implication in IL-12 production in response to R-848 in ovine cells. Indeed, when p38/MAPK inhibitor SB203580 was added to R-848 stimulated MLN cells, the level of IL-12 produced was reduced by 41.3±7.5% (p<0.005). Western blots showed that neonatal CD14^+^ cells displayed higher rates of phosphorylation than their adult counterparts 15 minutes after stimulation with R-848, these rates being even higher after 30 minutes (Fig. 8D).

## Discussion

Several studies have focused on the specific features of the neonatal response to TLR agonists. Most were performed on human cord blood cells or mouse spleen cells. In this study, we aimed to extend this knowledge to mesenteric lymph node cells, as they represent a key site not only for the induction of tolerance to food proteins but also for the induction of specific immune responses against intestinal pathogens. Moreover, they act as a firewall, preventing live commensal intestinal bacteria from penetrating the systemic immune system [34].

Our ELISA data show that MLN cells from newborn lambs have a strong IL-12 response to R-848. The antibodies used for the IL-12 ELISA cannot distinguish between the bioactive IL-12p70 from the IL-12p40 subunit. The p40 chain can be produced as monomer, associated to p19 chain to form IL-23 or as an antagonist IL-12p40 homodimer (IL-12p80). However, as we also observed high levels of IFNγ release by MLN neonatal cells that can be severely reduced by *in vitro* IL-12 neutralisation, this suggests that neonate produce more bioactive IL-12p70. Moreover, qRT-PCR and ELISA time courses confirmed that IL-12 production preceded IFNγ gene expression. We therefore focused on the reasons for this difference in IL-12 production between neonates and adults. CD14^+^ cells were identified, by intracellular staining and *in vitro* depletion, as the main population producing IL-12 in response to the *in vitro* stimulation of MLN cells with R-848.

In our study, MLN CD14^+^ cells co-expressed the myeloid markers CD11b and CD40, but not CD135 (Flt3). Electron microscopy showed these cells to have several characteristics of DCs, such as long dendritic processes, electron-dense granules and a well developed endosomal compartment. Ovine DCs have been well characterised in afferent lymph [23], [35] and when derived *in vitro* from bone marrow cells [30], but the various subsets present in lymph nodes have yet to be described. In PBMCs from humans and cattle, CD14^+^ cell depletion abolishes the IL-12 response to R-848 [17], [36]. Our results based on phenotypic and morphological characteristics of these cells suggest that CD14^+^ CD11b^+^ CD40^+^ CD135^−^ cells are a subpopulation of myeloid DCs that develop from blood monocytes independently of the Flt3 ligand.

The difference in the IL-12 response between neonatal and adult MLN cells may result from several factors, including a difference in the number of CD14^+^ cells, qualitative differences and/or environmental factors affecting the response of these cells. Higher or lower frequencies of immune cell populations in the blood or organs of neonates are commonly reported. As differences in the frequency of CD14^+^ cells among MLN cells may directly modify the amount of IL-12 produced in response to TLR stimulation, we analysed the proportions of these cells in both adults and neonates. CD14^+^ cells accounted for a higher proportion of MLN cells in neonates, and this proportion rapidly decreased with age, from 16% in one- to two-week-old animals to 10% in 20-day-old lambs and finally reaching 7% in adult animals.

This may directly contribute to the strong IL-12 response to R-848 of neonatal MLN cells. However, we also observed that isolated neonatal CD14^+^ cells were more responsive than their adult counterparts to imidazoquinoline compounds. R-848 binds TLR7 and TLR8, but the responses to this binding differ between animal species [32]. Our results, obtained with the more specific TLR7 and TLR8 agonists, CL087 and CL075, respectively, suggest that TLR8 was the principal receptor involved in the IL-12 response to R-848 in ovine cells, consistent with previous findings for cattle PBMCs [17]. This conclusion is supported by the preferential expression of TLR8, which is present at much higher levels than TLR7, on CD14^+^ MLN cells.

However, the level of TLR8 expression is similar on neonatal and adult CD14^+^ cells, suggesting that the difference in response to R-848 between neonates and adults depends on other differences affecting regulation by external or intracellular cytokines or by signalling events downstream from TLR8.

IL-12 production by MLN cells was tightly regulated by the regulatory cytokine IL-10. In mouse, neonatal splenic regulatory B cells (Breg) expressing CD5 have been shown to produce large amounts of IL-10 in response to the *in vivo* administration of TLR agonists that significantly decrease IL-12 production [13], [37]. Similarly, Booth *et al*. observed that sheep Peyer's patch cells displayed poor IFNγ, IFNα and IL-12 responses to CpG but secreted large amounts of IL-10. By depleting the CD5^−^ CD21^+^ B-cell population or neutralising IL-10, these authors obtained a significant increase in the CpG-induced IFNγ response, suggesting that IL-10 from this Breg cell population may regulate the immune response in Peyer's patches [38]. However, MLN cells in the same conditions produced five to 10 times more IL-12 than Peyer's patch cells. As we observed that only neonatal MLN cells produced low amounts of IL-10 in response to R-848, Breg are unlikely to be involved in the difference in cytokine response observed between neonates and adults in our experimental model. Moreover, we observed that the CD14^+^ population responsible for the strong IL-12 response in neonates also produced most, if not all of the low amount of IL-10 released after R-848 stimulation (data not shown). Treg constitute a population of adaptive immune system cells that can regulate both innate and adaptive immune responses by secreting cytokines (*e.g.* IL-10 and TGFβ1) and by cell-cell contact. Nevertheless, we observed that Foxp3 levels did not differ significantly between neonates and adults in the MLN and that rhTGFβ1, despite its cross-reactivity with ovine cells, could not abolish IL-12 secretion by MLN cells in response to R-848. We next investigated the expression of TLR-induced negative regulatory molecules SOCS in the response of neonatal and adult MLN cells to R-848. CD14^+^ MLN cells from neonates strongly upregulated IL-12/23 chain p40, which was much more abundant than in adults by 3 h after stimulation with R-848. This was associated with a concomitant upregulation of SOCS-1 and SOCS-3 mRNA. This suggests that these regulatory molecules (IL-10, SOCS-1 and SOCS-3) are produced to downregulate inflammatory signals induced strongly in neonatal but only poorly in adult CD14^+^ cells. We then investigated whether signalling events downstream from TLR8 ligation could account for the difference in IL-12 response between neonates and adults. The exposure of human neonatal or adult APCs to TLR8 agonists has been reported to induce the robust phosphorylation of p38/MAPK and the prolonged degradation of IκBα? resulting in the robust induction of Th1-type immune responses, including the production of IL-12 and the upregulation of costimulatory molecule CD40 [39]. The level of p38/MAPK phosphorylation rate was higher in neonatal CD14^+^ cells stimulated with R-848 than in their adult counterparts.

The rapid weakening of the strong IL-12 response to TLR8 stimulation, observed from 20 days onwards in the MLN, is due to the combination of both a change in the number of CD14^+^ cells and qualitative differences in downstream intracellular signalling pathways. Indeed, despite the production of IL-10 and the upregulation of SOCS-1 and SOCS-3 mRNA, neonatal CD14^+^ MLN cells still display higher levels of p38/MAPK phosphorylation than their adult counterparts. This stronger phosphorylation of p38/MAPK in neonates could result as a consequence of stronger downstream TLR8 activation signals or preferential phosphorylation of p38/MAPK in neonates. Whatever the reasons, as already reported in humans, imidazoquinoline compounds are also powerful stimulators of the innate immune system of neonatal lambs that could be considered for use in early mucosal vaccination strategies.

## Supporting Information

Table S1 Primers used for qRT-PCR analysis.
(0.04 MB DOC)
