# Supplementary material for: Cellular and Molecular Mechanisms Underlying the Strong Neonatal IL-12 Response of Lamb Mesenteric Lymph Node Cells to R-848
Source: PLoS One. 2010 Oct 28;5(10):e13705. doi: 10.1371/journal.pone.0013705 (PMC2965667; doi:10.1371/journal.pone.0013705)
Supplement: Table S1 — Primers used for qRT-PCR analysis. (0.04 MB DOC) [file pone.0013705.s001.doc]

**Table S1:** Primers used for qRT-PCR analysis

| **Target mRNA** | **5’ Primer** | **3’ Primer** | **PCR product (bp)** |
| --- | --- | --- | --- |
| **FoxP3** | 5’-GAAACAGCACATTCCCAGAGT-3’ | 5’-TGTGCAGGCTCAGGTTGTGG-3’ | 228 |
| **IL-12/IL-23p40** | 5’-AACCTGCAACTGAGACCACT-3’ | 5’-ATCCTTGTGGCATGTGACTT-3’ | 186 |
| **IL-12p35** | 5’-TGGGCATTGTCTGTCTTCTG-3’ | 5’-AGGGTTTCTGTGGCACAGTC-3’ | 224 |
| **IL-23p19** | 5’-ATGGGACATGTGGATCTACC-3’ | 5’-CTTGGAGTCTGCTCAGTTTC-3’ | 229 |
| **IL-10** | 5’-AGCAGCTGTACCCACTTCC-3’ | 5’-CAGCAGAGACTGGGTCAAC-3’ | 132 |
| **TLR7** | 5’-GGTCTCTCTGGATCTATATAC-3’ | 5’-AGAAATGATGACTATACCACACA-3’ | 146 |
| **TLR8** | 5’-TCTAGCCATCATCGACAACCTCA-3’ | 5’-CAAGTAGAATGCCGTTTTAAAATT-3’ | 112 |
| **SOCS-1** | 5'-AGCGTGAAGATGGCCTCGGG-3' | 5'-TTAATGCTGCGGTGTGCGGC-3' | 314 |
| **SOCS-3** | 5'-CCCACAGCAAGTTTCCCGCC-3' | 5'-CGAGCTGTCGCGGATGAGGA-3' | 215 |
| **HPRT** | 5’-AAACCAAAGATGGTCAAGGT-3’ | 5’-TCTTAGGCTTTGTATTTTGCTT-3’ | 200 |
